# Early Hospital Mortality among Adult Trauma Patients Significantly Declined between 1998-2011: Three Single-Centre Cohorts from Mumbai, India

**DOI:** 10.1371/journal.pone.0090064

**Published:** 2014-03-03

**Authors:** Martin Gerdin, Nobhojit Roy, Satish Dharap, Vineet Kumar, Monty Khajanchi, Göran Tomson, Li Felländer Tsai, Max Petzold, Johan von Schreeb

**Affiliations:** 1 Health Systems and Policy, Department of Public Health Sciences, Karolinska Institutet, Stockholm, Sweden; 2 Department of Surgery, Bhabha Atomic Research Centre Hospital, Mumbai, India; 3 School of Habitat, Tata Institute of Social Sciences, Mumbai, India; 4 Department of Surgery, Lokmanya Tilak Municipal Medical College and General Hospital, Mumbai, India; 5 Department of Surgery, Seth G. S. Medical College & King Edward Memorial Hospital, Mumbai, India; 6 Medical Management Centre, Department of Learning, Informatics, Management and Ethics, Karolinska Institutet, Stockholm, Sweden; 7 Division of Orthopedics and Biotechnology, Department of Clinical Science Intervention and Technology, Karolinska Institutet, Stockholm, Sweden; 8 Centre for Applied Biostatistics, Occupational and Environmental Medicine, Sahlgrenska Academy at University of Gothenburg, Gothenburg, Sweden; National Taiwan University, Taiwan

## Abstract

**Background:**

Traumatic injury causes more than five million deaths each year of which about 90% occur in low- and middle-income countries (LMIC). Hospital trauma mortality has been significantly reduced in high-income countries, but to what extent similar results have been achieved in LMIC has not been studied in detail. Here, we assessed if early hospital mortality in patients with trauma has changed over time in an urban lower middle-income setting.

**Methods:**

We conducted a retrospective study of patients admitted due to trauma in 1998, 2002, and 2011 to a large public hospital in Mumbai, India. Our outcome measure was early hospital mortality, defined as death between admission and 24-hours. We used multivariate logistic regression to assess the association between time and early hospital mortality, adjusting for patient case-mix. Injury severity was quantified using International Classification of Diseases-derived Injury Severity Score (ICISS). Major trauma was defined as ICISS<0.90.

**Results:**

We analysed data on 4189 patients out of which 86.5% were males. A majority of patients were between 15 and 55 years old and 36.5% had major trauma. Overall early hospital mortality was 8.9% in 1998, 6.0% in 2002, and 8.1% in 2011. Among major trauma patients, early hospital mortality was 13.4%, in 1998, 11.3% in 2002, and 10.9% in 2011. Compared to trauma patients admitted in 1998, those admitted in 2011 had lower odds for early hospital mortality (OR = 0.56, 95% CI = 0.41–0.76) including those with major trauma (OR = 0.57, 95% CI = 0.41–0.78).

**Conclusions:**

We observed a significant reduction in early hospital mortality among patients with major trauma between 1998 and 2011. Improved survival was evident only after we adjusted for patient case-mix. This finding highlights the importance of risk-adjustment when studying longitudinal mortality trends.

## Background

Traumatic injury is a major threat to global public health [1], [2]. More people die annually from traumatic injuries than from HIV/AIDS, tuberculosis, malaria, and obstetric conditions combined [1]. Over 90% of the five million annual deaths from traumatic injuries occur in low- and middle-income countries (LMIC) [3], [4]. A recent study estimated that almost two million lives could be saved each year, if hospital care for the injured, i.e. trauma care in LMIC can be improved and reach the same level as in high-income countries (HIC) [5]. However, it is currently unclear how such reduction can be achieved.

Although a large part of trauma mortality occurs at the injury-site and during pre-hospital transportation, around 30-50% of trauma mortality occurs in hospital [6], [7]. In HIC, hospital trauma mortality has been significantly reduced in recent years [8], [9]. Among the most important explanations for this reduction are implementation of trauma care systems and improved medical and surgical treatment [10], [11], including management of traumatic brain injury [12], haemorrhage control [13], and musculoskeletal injuries [14], [15]. These improvements in clinical care have been driven, in large part, by research progress, from the basic sciences to the systems and policy level.

Hospital trauma registers have played a key role in the advancement of patient-based research and trauma care in HIC [16], [17]. Trauma registers offer a unique opportunity to document patient characteristics and audit outcomes [18], thereby creating a platform for innovative clinical research. Moreover, trauma registers in HIC have been used to study longitudinal trends in hospital mortality [9]. Such studies are important as they form the basis for further research on factors underlying changes in hospital mortality. In contrast, few trauma registers exist in LMIC [19], [20], which limit the potential for studies of longitudinal trends in hospital mortality in these countries.

Studies from HIC have stressed the importance of risk-adjustment when comparing trauma hospital mortality rates between hospitals or over time [21], [22]. Risk-adjustment is commonly done by incorporating measures of patient case-mix including injury severity or mortality risk in the analysis [23]. Several different methods for assigning mortality risk exist but the evidence for which one to use is inconclusive [24]. Recent research from HIC has used the data-driven score International Classification of Disease (ICD) based Injury Severity Score (ICISS) to adjust for mortality risk [25], but there is no consensus regarding the appropriate approach in studies from LMIC. We adopted an explorative approach to risk-adjustment using ICISS, with the aim to assess if early hospital mortality in patients with trauma has changed over time in an urban lower middle-income setting.

## Methods

### Study design

**Figure 1:**
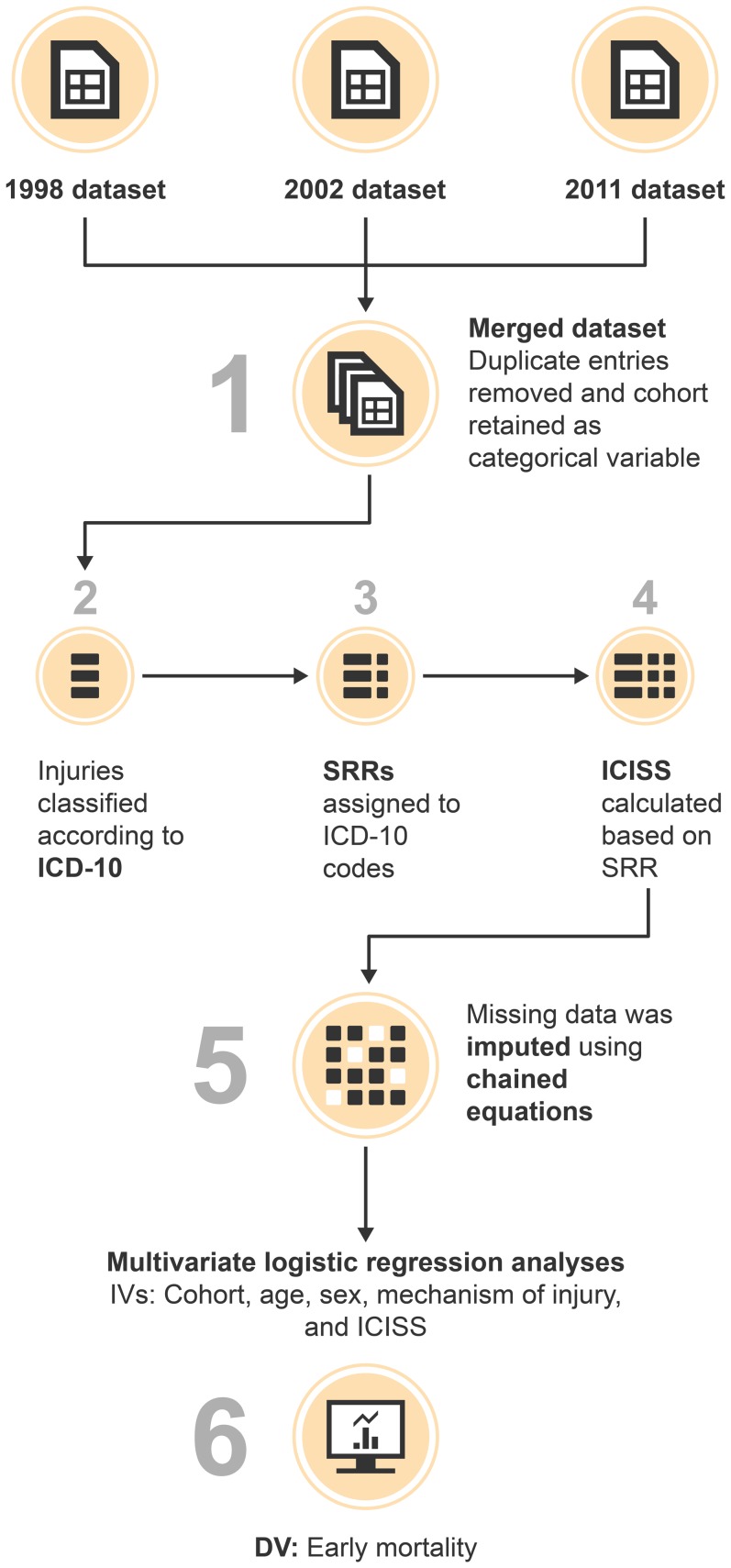
Flowchart providing a simplified outline of the study process. Abbreviations: DV Dependent Variable, ICD International Classification of Disease, ICISS ICD-derived Injury Severity Score, IV Independent Variables, SRR Survival Risk Ratio.

We conducted a retrospective study of patients presenting to Lokmanya Tilak Municipal General Hospital (LTMGH), Mumbai, India (Figure 1).

### Ethics statement

The institutional ethics committee of LTMGH approved the collation of the 2011 database, reference number IEC/22/10, and approved new analyses of all three dataset in an amendment to IEC/22/10. The ethics committee granted a waiver of consent.

### Setting

**Figure 2:**
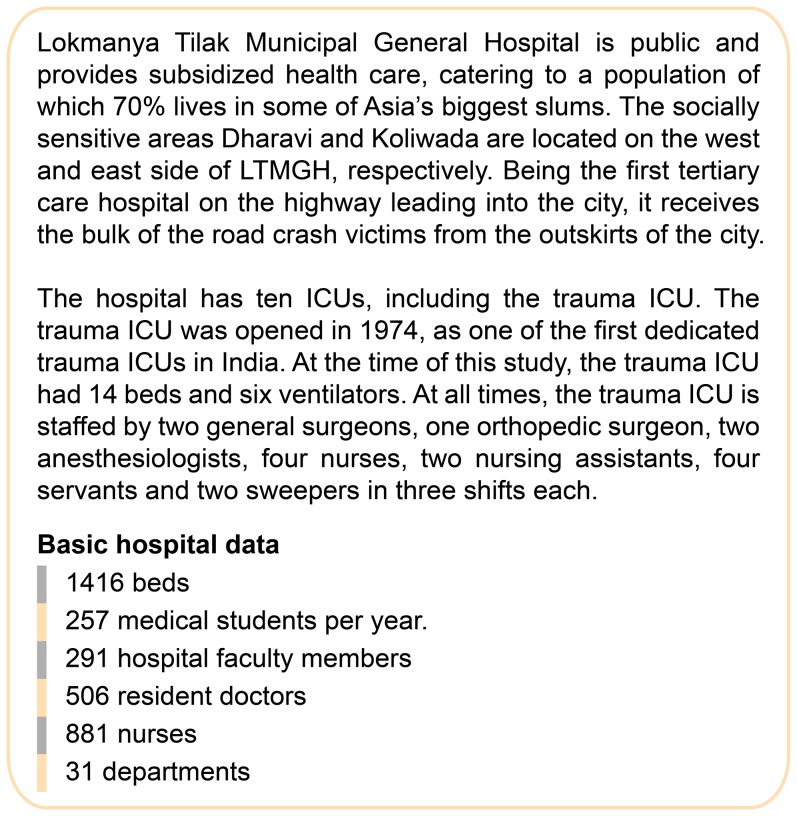
Characteristics of Lokmanya Tilak Municipal General Hospital. Abbreviations: ICU Intensive Care Unit.

India is the world's second most populous country, classified as a lower middle-income country by the World Bank [26]. In 2011, it was estimated that more than 500,000 people died from traumatic injuries in India [27]. In 2015, around 200,000 Indians are expected to die from road traffic injuries (RTI) [28]. Furthermore, in India, more than 50% of mortality from RTI occur in hospitals [29]. Mumbai is the most populous city in India, with more than18 million inhabitants [30]. Lokmanya Tilak Municipal General Hospital (Figure 2) is one of the four biggest public hospitals in Mumbai.

### Data

We retrospectively analysed three datasets of patient cohorts admitted to LTMGH trauma ward during 1998, 2002, and 2011 (Table 1). All three datasets were collated for previous research. At least one of the authors (NR, VK, or SD) of this study supervised collection of all three datasets. The three datasets were merged and duplicate entries were detected and removed using the automated approach suggested in STATA's FAQ [31].

**Table 1:** Characteristics of the 1998, 2002, and 2011 datasets.

	1998 dataset	2002 dataset	2011 dataset
**Purpose**	Research	Research	Research
**Time period (days)**	1 Jan 1998 - 31 Dec 1998 (365)	1 Aug 2001 - 31 May 2002 (304)	15 Oct 2010 - 31 Dec 2011 (443)
**Inclusion criteria**	Admitted to LTMGH trauma ICU	Admitted to LTMGH trauma ICU	Admitted to LTMGH trauma ICU with life or limb threatening injury^1^
**Number of patients included (after duplicates removed)**	2009 (2009)	1075 (1063)	1130 (1117)
**Data collection methods**	The surgical registrar of each unit collected data on patients admitted during his or her unit's duty.	The surgical registrar of each unit collected data on patients admitted during his or her unit's duty.	One dedicated data collector performed all data collection. Her timings in the trauma ward were randomized, covering eight hours each day, five days a week. She retrieved data from patients admitted during her off shift periods from intake forms.
**Categories of variables collected**	Demographics, mechanism of injury, level of consciousness, injuries, and early hospital mortality	Demographics, mechanism of injury, physiologic status, injuries, and early hospital mortality.	Demographics, mechanism of injury, physiologic status, length of stay in ICU and hospital, procedures and investigations performed, injuries, mortality, and time to death
**Sources for injury diagnoses**	Patient chart, x-rays, CT-scan findings, intraoperative findings and procedures	Patient chart, x-rays, CT-scan findings, intraoperative findings and procedures	Patient chart, x-rays, CT-scan findings, intraoperative findings and procedures
**Mode of injury recording**	Free text, categorized under head injury, orthopedic injury, chest injury, abdominal injury, and faciomaxillar inury	Free text, categorized under CT-findings, abdominal injury, pelvic injury, amputations, degloving injury, other orthopedic injury, and faciomaxillar injury	Free text
**Length of follow up**	24 hours	24 hours	Discharge or death

1 A list of injury mechanisms in combination with certain physiological signs (such as hypotension) was used to define life or limb threatening injury. Abbreviations: CT Computer Tomography, ICU Intensive Care Unit, ICU Intensive Care Unit, LTMGH Lokmanya Tilak Municipal General Hospital.

### Eligibility criteria

All non-duplicate observations were considered eligible. Observations falling outside of accepted value ranges were set to missing.

### Variables

Our outcome of interest was early hospital mortality, defined as death between admission and 24 hours. According to Haider et al. [23], risk adjusted outcome studies should account for sex, age, mechanism of injury, and physiological- and anatomical injury severity. We were able to include all except physiological injury severity as covariates. Age was defined as a categorical variable, with the conventionally used cut-offs <15, 15–55, and >55 years. Mechanism of injury was defined as a nominal variable, categorised as fall, railway injury (RI), road traffic injury (RTI), assault, other, and unknown.

### Anatomical injury severity

We used ICISS to quantify anatomical injury severity [32]. Our rationale was that ICISS has been shown to perform well compared to the more conventional Injury Severity Score (ISS) and other established injury severity measures such as Trauma and Injury Severity Score (TRISS) [33], [34], while also being easily computed using ICD-codes. To calculate ICISS, each ICD-code is assigned a survival risk ratio (SRR). Each SRR is equal to the proportion of patients who survived with a specific ICD-code in a reference population. In studies with large samples, the study sample is often used as the reference population [35], [36]. Smaller studies may use published SRRs from a larger, similar, reference population [37]. In literature searches, we found no published SRRs from a LMIC trauma population and therefore we decided to use our own sample as the reference population. Because our sample was small, this analysis should be considered explorative and we are well aware of the same-sample bias that potentially is introduced using this approach.

MG coded all patients' injuries using ICD-10: 2010 edition, down to the fourth level. We calculated SRRs by dividing the number of survivors with a specific ICD-code with the total number of patients with the same ICD-code. This was done separately for each cohort to account for potential differences in factors such as transport time and care that might have influenced outcomes in the three time periods. We used the ICISS1 version of ICISS, which means that for overall scoring of patients' injury severity we counted only the worst injury (lowest SRR). We chose ICISS1 because this version has been showed to have a higher predictive value compared to versions that take into account all of a patient's injuries [38]. We defined major trauma as ICISS<0.9 [39].

### Statistical methods

We used STATA (STATA 12, StataCorp, Texas) for statistical analyses. Where applicable, a significance level of 5% and a confidence level of 95% were used. Sample characteristics are reported for each cohort. Continuous variables are reported using their median, inter-quartile range, and range because they were found to be non-normally distributed. Categorical variables are reported as proportions.

We assessed the univariate associations between covariates and early hospital mortality using logistic regression and report their unadjusted odd-ratios (OR). We used multivariate logistic regression to evaluate the association between time and early hospital mortality. Dummy variables were created for the three cohorts and the 2002 and 2011 cohorts were included in the logistic regression model and compared early hospital mortality with the early hospital mortality in 1998. Age, sex, mechanism of injury, and ICISS were included as covariates [23]. We conducted 13 subgroup analyses and assessed early hospital mortality trends separately in patients with and without major trauma, males, females, each age category, and in patients with history of fall, railway injury, and road traffic injury.

We used multiple imputation using chained equations to handle missing data. For each variable with missing data, we assessed the association between the probability of missing data and early hospital mortality using logistic regression. We found no significant associations, i.e. P-value>0.05 in all analyses, and hence deemed multiple imputation as appropriate. Each cohort was imputed separately. We specified our imputation model to impute sex using logistic regression, and age and mechanism of injury using multinominal logistic regression. We performed 20 imputations to achieve stable estimates.

## Results

**Figure 3:**
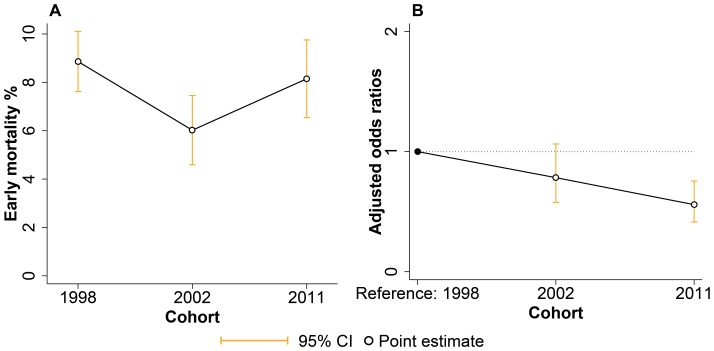
Unadjusted and adjusted comparison of early hospital mortality across cohorts. **A.** Unadjusted early hospital mortality rates across cohorts. **B.** Odds ratios for the 2002 and 2011 cohorts when treated as indicator variables in a multivariate logistic regression analysis with early hospital mortality as the dependent variable. The model was adjusted for sex, age, mechanism of injury and ICISS. Abbreviations: ICD International Classification of Disease, ICISS ICD-derived Injury Severity Score.

We analysed data on 4189 patients (Table 2), out of which 86.5% were male. Road traffic injury was the most common mechanism of injury across cohorts, followed by railway injury and falls. Out of all patients, 81.5% were between 15 and 55 years old. Early hospital mortality among all patients was 7.9% (Figure 3A), the median ICISS was 0.93, and 36.5% had major trauma (ICISS<0.9). The early hospital mortality rate among major trauma patients was 12.2% overall and 13.4%, 11.3%, and 10.9% in 1998, 2002, and 2011 respectively.

**Table 2:** Patient characteristics*.

	1998	2002	2011	All cohorts
**Number of patients**	2009	1063	1117	4189
**Males %** ^α^	87.3	83.9	87.7	86.5
**Age in years %** ^β^				
<15	9.8	13.1	13.0	11.4
15–55	84.9	78.1	78.1	81.5
>55	5.3	8.8	8.9	7.1
**Mechanism of injury %** ^γ^				
Fall	24.0	26.5	24.4	24.8
Railway injury	27.5	22.6	26.9	26.1
Road traffic injury	37.7	38.7	31.5	36.3
Assault	9.0	8.3	13.4	10.0
Other	0.7	2.8	2.7	1.1
Unknown	1.0	1.1	3.9	1.8
**Median ICISS (IQR:range)**	0.96 (0.87–0.99:0.00–1.00)	0.97 (0.93–0.98:0.00–1.00)	0.89 (0.86–0.93:0.00–1.00)	0.93 (0.88–0.98:0.00–1.00)
**Major trauma %**	37.6	15.8	54.2	36.5
**Early hospital mortality %**	8.9	6.0	8.1	7.9

*Values are expressed as medians (IQR:range) or proportions (%) where appropriate. ^α^0.1% missing data. ^β^5.4% missing data. ^γ^<0.1% missing data. Abbreviations: ICD International Classification of Disease, ICISS ICD-derived Injury Severity Score, IQR Inter Quartile Range.

### Univariate analysis

Our univariate logistic regression analyses (Table 3) showed that patients over 55 years of age had significantly higher odds of early hospital mortality compared to patients younger than 15 years of age (OR = 1.81, P-value = 0.026, 95% CI = 1.07–3.05). Patients with railway injury (OR = 3.21, P-value<0.001, 95% CI = 2.26–4.57), road traffic injury (OR = 1.78, P-value = 0.002, 95% CI = 1.24–2.55), or an unknown mechanism of injury (OR = 3.04, P-value = 0.004, 95% CI = 1.42–6.51) had significantly higher odds of early hospital mortality compared to patients with fall. Every 0.01 unit increase in ICISS was significantly associated with lower odds of early hospital mortality (OR = 0.95, P-value<0.001, 95% CI = 0.94–0.96), and having major trauma was significantly associated with higher odds of early hospital mortality (OR = 2.44, P-value<0.001, 95% CI = 1.93–3.09).

**Table 3:** Results from univariate logistic regression assessing associations between patient characteristics and early hospital mortality.

	Complete case analysis		Imputed values	
	OR (95% CI)	P-value	OR (95% CI)	P-value
**Male**	1.13 (0.79–1.60)	0.510	1.15 (0.82–1.62)	0.417
**Age in years**				
Reference: <15	1.00	.	1.00	.
15–55	1.17 (0.79–1.73)	0.439	1.17 (0.80–1.72)	0.420
>55	1.81 (1.07–3.05)	0.026	1.82 (1.09–3.05)	0.022
**Mechanism of injury**				
Reference: Fall	1.00	.	1.00	.
Railway injury	3.21 (2.26–4.57)	<0.001	3.18 (2.27–4.45)	<0.001
Road traffic injury	1.78 (1.24–2.55)	0.002	1.74 (1.23–2.45)	0.002
Assault	0.48 (0.23–1.00)	0.050	0.45 (0.22–0.93)	0.032
Other	1.22 (0.28–5.22)	0.792	0.96 (0.23–4.07)	0.954
Unknown	3.04 (1.42–6.51)	0.004	3.12 (1.51–6.45)	0.002
**ICISS per 0.01 increase**	0.95 (0.94–0.96)	<0.001	0.95 (0.94–0.96)	<0.001
**Major trauma**	2.44 (1.93–3.09)	<0.001	2.37 (1.89–2.97)	<0.001

Abbreviations: CI Confidence Interval, ICD International Classification of Disease, ICISS ICD-derived Injury Severity Score, OR Odds Ratio.

### Multivariate analysis

Compared to 1998, the multivariate logistic regression model showed lower adjusted odds of early hospital mortality in 2002 (OR = 0.78, P-value = 0.118, 95% CI = 0.58–1.06) and significantly lower odds in 2011 (OR = 0.56, P-value<0.001, 95% CI = 0.41–0.75), (Table 4, Figure 3B). Males did not have significantly higher odds of early hospital mortality compared to females. There was no significant difference in odds of early hospital mortality difference between either older (>55 years) or younger (15–55 years) compared to the youngest (<15 years) patients. Railway injury (OR = 2.85, P-value<0.001, 95% CI = 1.98–4.10) and road traffic injury (OR = 1.54, P-value = 0.021, 95% CI = 1.07–2.21) retained largely the same effect sizes and remained significantly associated with early hospital mortality in multivariate analysis. Assault was significantly associated with lower odds of early mortality (OR = 0.46, P-value = 0.038, 95% CI = 0.21–0.95). Also, every 0.01 unit increase in ICISS remained significantly associated with lower odds of early hospital mortality (OR = 0.95, P-value<0.001, 95% CI = 0.94–0.96). Model estimates for each cohort analysed separately are available as Table S1-S3.

**Table 4:** Multivariate logistic regression model parameters.

	Complete case analysis		Imputed values	
	OR (95% CI)	P-value	OR (95% CI)	P-value
**Cohort**				
Reference: 1998	1.00	.	1.00	.
2002	0.64 (0.45–0.92)	0.015	0.78 (0.58–1.06)	0.118
2011	0.56 (0.42–0.76)	<0.001	0.56 (0.41–0.75)	<0.001
**Age in years**				
Reference: <15	1.00	.	1.00	.
15–55	0.71 (0.46–1.09)	0.116	0.71 (0.46–1.08)	0.111
>55	1.38 (0.79–2.39)	0.254	1.32 (0.76–2.29)	0.322
**Male**	1.15 (0.78–1.70)	0.472	1.17 (0.80–1.70)	0.416
**Mechanism of injury**				
Reference: Fall	1.00	.	1.00	.
Railway injury	2.78 (1.90–4.05)	<0.001	2.85 (1.98–4.10)	<0.001
Road traffic injury	1.52 (1.05–2.22)	0.028	1.54 (1.07–2.21)	0.021
Assault	0.46 (0.21–1.00)	0.050	0.44 (0.21–0.95)	0.038
Other	1.36 (0.31–5.96)	0.683	0.99 (0.23–4.30)	0.994
Unknown	2.62 (1.14–6.01)	0.023	2.86 (1.30–6.28)	0.009
**ICISS per 0.01 increase**	0.95 (0.94–0.96)	<0.001	0.95 (0.94–0.96)	<0.001

Abbreviations: CI Confidence Interval, ICD International Classification of Disease, ICISS ICD-derived Injury Severity Score, OR Odds Ratio.

### Subgroup analysis

**Figure 4:**
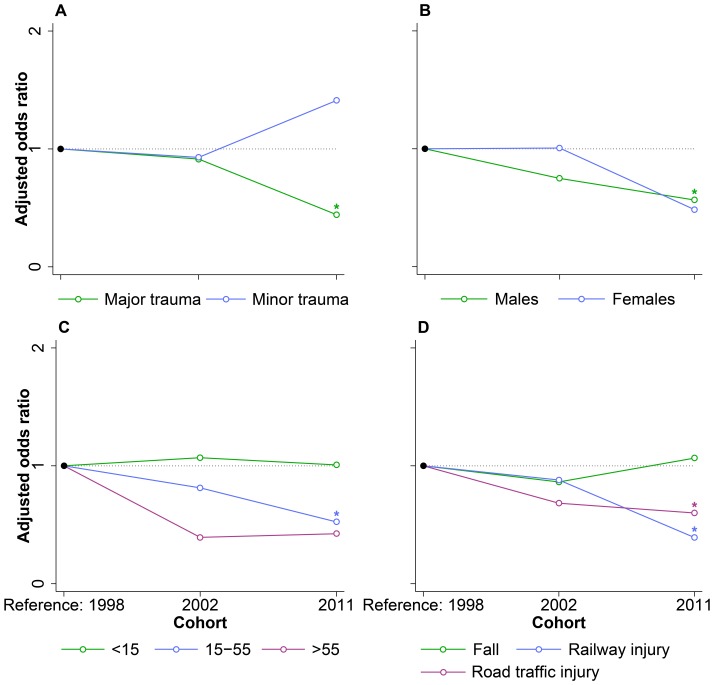
Comparison of early hospital mortality odds ratios in subgroup analyses. Odds ratios are early hospital mortality odds ratios. **A.** Major trauma was defined as ICISS<0.9. The model was adjusted for sex, age, mechanism of injury, and ICISS. **B.** The model was adjusted for age, mechanism of injury, and ICISS. **C.** The model was adjusted for sex, mechanism of injury, and ICISS. **D.** The model was adjusted for sex, age, and ICISS. Abbreviations: ICD International Classification of Disease, ICISS ICD-derived Injury Severity Score. *P-value<0.05, compared to the reference category.

Among patients with major trauma, the odds of early hospital mortality were significantly lower in 2011 (OR = 0.44, P-value<0.001, 95% CI = 0.30–0.66) compared to 1998 (Figure 4A). Males had significantly lower odds of early hospital mortality in 2011 (OR = 0.57, P-value<0.001, 95% CI = 0.41–0.78) compared to 1998 (Figure 4B). In patients between 15 and 55 years of age the odds of early hospital mortality were significantly lower in 2011 (OR = 0.52, P-value<0.001, 95% CI = 0.37–0.74) compared to 1998 (Figure 4C). For patients with railway injury the odds were significantly lower in 2011 (OR = 0.39, P-value<0.001, 95% CI = 0.24–0.64) compared to 1998 (Figure 4D). Patients with road traffic injury had significantly lower odds in 2011 (OR = 0.60, P-value = 0.047, 95% CI = 0.36–1.00) compared to 1998. Full model estimates for all subgroups, including non-significant results, are available as Table S4-13.

## Discussion

Our study showed that early hospital mortality in trauma patients decreased over a 13-year period in an urban lower middle-income setting. This trend was not observed in unadjusted analysis and our study therefore highlights the importance of risk-adjustment when comparing mortality rates over time. However, we used an explorative approach to compute ICISS as a measure of anatomical injury severity and this should be kept in mind when interpreting our results. The observed reduction in early hospital mortality was most pronounced in patients with major trauma, while no significant changes could be detected in patients with minor trauma. Similar trends as the one shown here have been observed for other major conditions in the same geographical area. For example, the infant mortality rate and under-five mortality rate in Maharashtra dropped by 25% and 34% respectively between 1992 and 2006 [40]. Similarly, Maharashtra's maternal mortality rate dropped by 23% between 1998 and 2009 [41], [42]. It is important to note that these figures are not risk-adjusted.

The implementation of trauma systems has been widely claimed as a major reason for reduced trauma mortality over time in HIC [11], [22], [43], [44]. The trauma system concept originates from the designation of dedicated trauma centres in the US some 40 years ago [45]. Today such systems ideally include streamlined preventive, prehospital, hospital, and rehabilitative measures [8]. Interestingly, the reduction in early hospital mortality observed in our study has occurred in the absence of a comprehensive trauma system. On the hospital level, LTMGH does employ a trauma team model, and one hypothetical explanation for the observed trend might be a maturation of trauma care efforts at institutional level, with improved equipment and enforced education of residents according to advanced trauma life support principles.

A functioning prehospital system is generally considered a vital component of a modern trauma system and improvements in prehospital triage and care may influence overall mortality rates. A recent systematic review found that the implementation of prehospital systems in LMIC has led to reduced overall mortality rates [46]. However, there is no organised prehospital system in Mumbai and most trauma patients arrive to hospital in taxis or are brought by the police [47]. Ambulances are used almost exclusively for inter-hospital transfers and are largely used as transport vehicles without any resuscitation equipment [47]. Therefore, the reduced odds of early hospital mortality can most likely not be attributed to improvements in prehospital care, but rather improved hospital trauma care.

A high proportion of the patients in this study were males. This finding is consistent with other research from LMIC [48]–[50]. Interestingly, the proportion of females seems to be higher in HIC trauma than in our study population [9], [51]. This discrepancy might be partly due to more women involved in work outside of the home in HIC compared to LMIC. Lastly, we found that RTI, falls, and RI were the most common mechanisms of injury. Although RTI and falls are major causes of trauma around the world, RI appears to be rather unique for Mumbai, and thus interesting to explore further as a subset of the Mumbai trauma population.

### Methodological considerations and limitations

First, the difference in inclusion criteria between the three cohorts is a major limitation. To adjust for this limitation and to minimize bias, we adjusted our analyses for case-mix and also conducted several subgroup analyses. Second, we used ICISS to adjust for anatomical injury severity and used our own data to calculate SRRs and generate finals scores. As we highlighted in the methodology section this approach might have introduced a same-sample bias that in part can explain the good fit of ICISS. Our approach should therefore be considered explorative in nature. However, we argue that this was a more sensible approach compared to using SRRs derived from a completely different trauma population. Third, we analysed available datasets and were therefore not able to choose our time-points. Unfortunately, retrospective inquires into patient files spanning more than ten years back constitute a more or less impossible undertaking as files are paper based and stored under variable conditions over time. Finally, the data did not allow us to explore outcome measures beyond mortality between admission and 24 hours. We do acknowledge that later mortality and functional outcomes are important and should be the focus of prospective studies in the same setting.

### Conclusions

In our study, we observed a significant reduction in early trauma mortality among patients with major trauma between 1998 and 2011. Improved survival was evident after adjusting for patient case-mix and this finding therefore highlights the importance of risk-adjustment in studies of mortality over time. Furthermore, as one of few studies from an urban lower-middle income setting spanning more than ten years, we show that survival has improved despite the absence of a trauma system and functioning prehospital organisation.

## Supporting Information

Table S1 Multivariate logistic regression model parameters, 1998 cohort analysed separately.
(PDF)

Table S2 Multivariate logistic regression model parameters, 2002 cohort analysed separately.
(PDF)

Table S3 Multivariate logistic regression model parameters, 2011 cohort analysed separately.
(PDF)

Table S4 Multivariate logistic regression model parameters, patients with major trauma analysed separately.
(PDF)

Table S5 Multivariate logistic regression model parameters, patients with minor trauma analysed separately.
(PDF)

Table S6 Multivariate logistic regression model parameters, males analysed separately.
(PDF)

Table S7 Multivariate logistic regression model parameters, females analysed separately.
(PDF)

Table S8 Multivariate logistic regression model parameters, age<15 years analysed separately.
(PDF)

Table S9 Multivariate logistic regression model parameters, age 15–55 years analysed separately.
(PDF)

Table S10 Multivariate logistic regression model parameters, age>55 years analysed separately.
(PDF)

Table S11 Multivariate logistic regression model parameters, patients with fall analysed separately.
(PDF)

Table S12 Multivariate logistic regression model parameters, patients with railway injury analysed separately.
(PDF)

Table S13 Multivariate logistic regression model parameters, patients with road traffic injury analysed separately.
(PDF)
