# Correction: Early Hospital Mortality among Adult Trauma Patients Significantly Declined between 1998-2011: Three Single-Centre Cohorts from Mumbai, India

**DOI:** 10.1371/journal.pone.0099694

**Published:** 2014-06-02

**Authors:** The PLOS ONE Staff

The seventh author’s name is spelled incorrectly. The correct name is Li Felländer-Tsai.
