# Supplementary material for: Early Hospital Mortality among Adult Trauma Patients Significantly Declined between 1998-2011: Three Single-Centre Cohorts from Mumbai, India
Source: PLoS One. 2014 Mar 3;9(3):e90064. doi: 10.1371/journal.pone.0090064 (PMC3940776; doi:10.1371/journal.pone.0090064)
Supplement: Table S1 — Multivariate logistic regression model parameters, 1998 cohort analysed separately. (PDF) [file pone.0090064.s001.pdf]

Table S1. Multivariate logistic regression model parameters, 1998 cohort analysed separately

	**Complete case analysis**		**Imputed values**	
	**OR (95% CI)**	**P-value**	**OR (95% CI)**	**P-value**
**Age in years**				
Reference: <15	1.00	.	1.00	.
15-55	0.91 (0.48-1.72)	0.761	0.91 (0.48-1.72)	0.760
>55	2.15 (0.97-4.78)	0.061	2.15 (0.97-4.78)	0.061
**Male**	1.19 (0.71-2.01)	0.511	1.19 (0.71-2.01)	0.511
**Mechanism of injury**				
Reference: Fall	1.00	.	1.00	.
Railway injury	3.52 (2.11-5.89)	<0.001	3.52 (2.11-5.89)	<0.001
Road traffic injury	1.86 (1.11-3.11)	0.019	1.85 (1.11-3.11)	0.019
Assault	0.41 (0.12-1.40)	0.156	0.41 (0.12-1.40)	0.153
Other	3.14 (0.65-15.18)	0.154	3.14 (0.65-15.17)	0.154
Unknown	3.44 (0.88-13.36)	0.075	3.30 (0.86-12.76)	0.083
**ICISS**	0.95 (0.94-0.97)	<0.001	0.95 (0.94-0.97)	<0.001

Abbreviations: CI Confidence Interval, ICD International Classification of Disease, ICISS ICD-derived Injury Severity Score, OR Odds Ratio
