# Supplementary material for: Early Hospital Mortality among Adult Trauma Patients Significantly Declined between 1998-2011: Three Single-Centre Cohorts from Mumbai, India
Source: PLoS One. 2014 Mar 3;9(3):e90064. doi: 10.1371/journal.pone.0090064 (PMC3940776; doi:10.1371/journal.pone.0090064)
Supplement: Table S2 — Multivariate logistic regression model parameters, 2002 cohort analysed separately. (PDF) [file pone.0090064.s002.pdf]

Table S2. Multivariate logistic regression model parameters, 2002 cohort analysed separately

	Complete case analysis		Imputed values	
	**OR (95% CI)**	**P-value**	**OR (95% CI)**	**P-value**
**Age in years**				
Reference: <15	1.00	.	1.00	.
15-55	0.72 (0.25-2.05)	0.535	0.71 (0.27-1.88)	0.489
>55	0.90 (0.22-3.58)	0.877	0.84 (0.21-3.31)	0.806
**Male**	0.77 (0.34-1.77)	0.543	0.94 (0.45-1.94)	0.861
**Mechanism of injury**				
Reference: Fall	1.00	.	1.00	.
Railway injury	3.49 (1.31-9.29)	0.013	3.53 (1.56-7.95)	0.002
Road traffic injury	1.58 (0.60-4.16)	0.351	1.56 (0.70-3.46)	0.272
Assault	0.99 (0.19-5.04)	0.987	0.72 (0.15-3.47)	0.686
Other	1.00	.	1.00	.
Unknown	1.00	.	2.71 (0.31-23.61)	0.367
**ICISS**	0.95 (0.90-1.00)	0.066	0.94 (0.91-0.98)	0.002

Abbreviations: CI Confidence Interval, ICD International Classification of Disease, ICISS ICD-derived Injury Severity Score, OR Odds Ratio
