# Supplementary material for: Early Hospital Mortality among Adult Trauma Patients Significantly Declined between 1998-2011: Three Single-Centre Cohorts from Mumbai, India
Source: PLoS One. 2014 Mar 3;9(3):e90064. doi: 10.1371/journal.pone.0090064 (PMC3940776; doi:10.1371/journal.pone.0090064)
Supplement: Table S3 — Multivariate logistic regression model parameters, 2011 cohort analysed separately. (PDF) [file pone.0090064.s003.pdf]

**Table S3.** Multivariate logistic regression model parameters, 2011 cohort analysed separately

| | **Complete case analysis** | | **Imputed values** | |
|---|---|---|---|---|
| | **OR (95% CI)** | **P-value** | **OR (95% CI)** | **P-value** |
| **Age in years** | | | | |
| Reference: <15 | 1.00 | . | 1.00 | . |
| 15-55 | 0.52 (0.24-1.12) | 0.096 | 0.53 (0.25-1.13) | 0.100 |
| >55 | 1.01 (0.39-2.63) | 0.990 | 1.01 (0.39-2.65) | 0.980 |
| **Male** | 1.51 (0.66-3.47) | 0.327 | 1.49 (0.65-3.42) | 0.341 |
| **Mechanism of injury*** | | | | |
| Reference: Fall | 1.00 | . | 1.00 | . |
| Railway injury | 1.58 (0.77-3.22) | 0.212 | 1.60 (0.78-3.27) | 0.197 |
| Road traffic injury | 1.07 (0.53-2.17) | 0.842 | 1.09 (0.54-2.21) | 0.801 |
| Assault | 0.31 (0.08-1.16) | 0.082 | 0.31 (0.08-1.15) | 0.081 |
| Other | 2.11 (0.68-6.58) | 0.197 | 2.15 (0.69-6.69) | 0.187 |
| **ICISS** | 0.94 (0.93-0.95) | <0.001 | 0.94 (0.93-0.95) | <0.001 |

*Unknown category dropped because of no observations. Abbreviations: CI Confidence Interval, ICD International Classification of Disease, ICISS ICD-derived Injury Severity Score, OR Odds Ratio
