# Supplementary material for: Early Hospital Mortality among Adult Trauma Patients Significantly Declined between 1998-2011: Three Single-Centre Cohorts from Mumbai, India
Source: PLoS One. 2014 Mar 3;9(3):e90064. doi: 10.1371/journal.pone.0090064 (PMC3940776; doi:10.1371/journal.pone.0090064)
Supplement: Table S4 — Multivariate logistic regression model parameters, patients with major trauma analysed separately. (PDF) [file pone.0090064.s004.pdf]

Table S4. Multivariate logistic regression model parameters, patients with major trauma analysed separately

	Complete case analysis		Imputed values	
	**OR (95% CI)**	**P-value**	**OR (95% CI)**	**P-value**
**Cohort**				
Reference: 1998	1.00	.	1.00	.
2002	0.78 (0.41-1.50)	0.463	0.91 (0.53-1.59)	0.748
2011	0.45 (0.30-0.67)	<0.001	0.44 (0.30-0.66)	<0.001
**Male**	1.22 (0.70-2.13)	0.486	1.16 (0.68-2.01)	0.584
**Age in years**				
Reference: <15	1.00	.	1.00	.
15-55	0.74 (0.37-1.47)	0.386	0.76 (0.38-1.51)	0.436
>55	1.51 (0.64-3.55)	0.343	1.51 (0.64-3.54)	0.343
**Mechanism of injury**				
Reference: Fall	1.00	.	1.00	.
Railway injury	3.28 (1.82-5.90)	<0.001	3.42 (1.90-6.14)	<0.001
Road traffic injury	1.35 (0.74-2.47)	0.324	1.38 (0.76-2.51)	0.289
Assault	0.60 (0.18-1.99)	0.404	0.59 (0.18-1.96)	0.388
Other	1.46 (0.17-12.44)	0.731	1.05 (0.13-8.80)	0.964
Unknown	3.70 (1.24-11.07)	0.019	3.82 (1.28-11.41)	0.017
**ICISS**	0.93 (0.92-0.95)	<0.001	0.93 (0.92-0.95)	<0.001

Abbreviations: CI Confidence Interval, ICD International Classification of Disease, ICISS ICD-derived Injury Severity Score, OR Odds Ratio
