# Supplementary material for: Early Hospital Mortality among Adult Trauma Patients Significantly Declined between 1998-2011: Three Single-Centre Cohorts from Mumbai, India
Source: PLoS One. 2014 Mar 3;9(3):e90064. doi: 10.1371/journal.pone.0090064 (PMC3940776; doi:10.1371/journal.pone.0090064)
Supplement: Table S5 — Multivariate logistic regression model parameters, patients with minor trauma analysed separately. (PDF) [file pone.0090064.s005.pdf]

Table S5. Multivariate logistic regression model parameters, patients with minor trauma analysed separately

	Complete case analysis		Imputed values	
	**OR (95% CI)**	**P-value**	**OR (95% CI)**	**P-value**
**Cohort**				
Reference: 1998	1.00	.	1.00	.
2002	0.75 (0.48-1.18)	0.219	0.93 (0.63-1.37)	0.712
2011	1.49 (0.86-2.60)	0.157	1.41 (0.82-2.44)	0.215
**Male**	1.14 (0.66-1.97)	0.644	1.22 (0.72-2.07)	0.470
**Age in years**				
Reference: <15	1.00	.	1.00	.
15-55	0.75 (0.42-1.32)	0.318	0.73 (0.42-1.28)	0.277
>55	1.51 (0.72-3.16)	0.278	1.39 (0.66-2.94)	0.390
**Mechanism of injury**				
Reference: Fall	1.00	.	1.00	.
Railway injury	2.63 (1.55-4.47)	<0.001	2.65 (1.60-4.38)	<0.001
Road traffic injury	2.12 (1.29-3.48)	0.003	2.04 (1.27-3.27)	0.003
Assault	0.43 (0.15-1.26)	0.126	0.39 (0.13-1.13)	0.082
Other	1.21 (0.15-9.47)	0.855	0.89 (0.12-6.83)	0.908
Unknown	1.62 (0.36-7.22)	0.528	2.12 (0.61-7.40)	0.240
**ICISS**	1.15 (1.07-1.24)	<0.001	1.13 (1.06-1.22)	<0.001

Abbreviations: CI Confidence Interval, ICD International Classification of Disease, ICISS ICD-derived Injury Severity Score, OR Odds Ratio
