# Supplementary material for: Early Hospital Mortality among Adult Trauma Patients Significantly Declined between 1998-2011: Three Single-Centre Cohorts from Mumbai, India
Source: PLoS One. 2014 Mar 3;9(3):e90064. doi: 10.1371/journal.pone.0090064 (PMC3940776; doi:10.1371/journal.pone.0090064)
Supplement: Table S6 — Multivariate logistic regression model parameters, males analysed separately. (PDF) [file pone.0090064.s006.pdf]

Table S6. Multivariate logistic regression model parameters, males analysed separately

	**Complete case analysis**		**Imputed values**	
	**OR (95% CI)**	**P-value**	**OR (95% CI)**	**P-value**
**Cohort**				
Reference: 1998	1.00	.	1.00	.
2002	0.59 (0.40-0.88)	0.009	0.75 (0.54-1.04)	0.088
2011	0.57 (0.41-0.78)	0.001	0.57 (0.41-0.78)	<0.001
**Age in years**				
Reference: <15	1.00	.	1.00	.
15-55	0.73 (0.44-1.20)	0.208	0.73 (0.44-1.19)	0.200
>55	1.68 (0.89-3.15)	0.109	1.59 (0.85-2.96)	0.143
**Mechanism of injury**				
Reference: Fall	1.00	.	1.00	.
Railway injury	2.73 (1.82-4.10)	<0.001	2.75 (1.86-4.06)	<0.001
Road traffic injury	1.54 (1.03-2.32)	0.037	1.52 (1.03-2.25)	0.036
Assault	0.41 (0.18-0.97)	0.042	0.39 (0.17-0.91)	0.028
Other	1.53 (0.34-6.79)	0.579	1.09 (0.25-4.76)	0.913
Unknown	2.67 (1.15-6.19)	0.022	2.86 (1.29-6.36)	0.010
**ICISS**	0.95 (0.94-0.96)	<0.001	0.95 (0.94-0.96)	<0.001

Abbreviations: CI Confidence Interval, ICD International Classification of Disease, ICISS ICD-derived Injury Severity Score, OR Odds Ratio
