# Supplementary material for: Early Hospital Mortality among Adult Trauma Patients Significantly Declined between 1998-2011: Three Single-Centre Cohorts from Mumbai, India
Source: PLoS One. 2014 Mar 3;9(3):e90064. doi: 10.1371/journal.pone.0090064 (PMC3940776; doi:10.1371/journal.pone.0090064)
Supplement: Table S7 — Multivariate logistic regression model parameters, females analysed separately. (PDF) [file pone.0090064.s007.pdf]

Table S7. Multivariate logistic regression model parameters, females analysed separately

	Complete case analysis		Imputed values	
	**OR (95% CI)**	**P-value**	**OR (95% CI)**	**P-value**
**Cohort**				
Reference: 1998	1.00	.	1.00	.
2002	0.94 (0.39-2.27)	0.895	1.01 (0.44-2.28)	0.987
2011	0.48 (0.18-1.29)	0.147	0.48 (0.18-1.30)	0.150
**Age in years**				
Reference: <15	1.00	.	1.00	.
15-55	0.67 (0.27-1.68)	0.395	0.66 (0.26-1.66)	0.378
>55	0.63 (0.18-2.18)	0.463	0.62 (0.18-2.12)	0.441
**Mechanism of injury**				
Reference: Fall	1.00	.	1.00	.
Railway injury	3.27 (1.14-9.37)	0.027	3.77 (1.33-10.68)	0.012
Road traffic injury	1.43 (0.54-3.84)	0.474	1.61 (0.61-4.26)	0.333
Assault	0.95 (0.13-7.05)	0.959	1.01 (0.14-7.46)	0.995
Other	1.00	.	1.00	.
Unknown	1.00	.	1.00	.
**ICISS**	0.94 (0.91-0.97)	<0.001	0.94 (0.91-0.97)	<0.001

Abbreviations: CI Confidence Interval, ICD International Classification of Disease, ICISS ICD-derived Injury Severity Score, OR Odds Ratio
