# Supplementary material for: Early Hospital Mortality among Adult Trauma Patients Significantly Declined between 1998-2011: Three Single-Centre Cohorts from Mumbai, India
Source: PLoS One. 2014 Mar 3;9(3):e90064. doi: 10.1371/journal.pone.0090064 (PMC3940776; doi:10.1371/journal.pone.0090064)
Supplement: Table S8 — Multivariate logistic regression model parameters, age<15 years analysed separately. (PDF) [file pone.0090064.s008.pdf]

Table S8. Multivariate logistic regression model parameters, age<15 years analysed separately

	Complete case analysis		Imputed values	
	**OR (95% CI)**	**P-value**	**OR (95% CI)**	**P-value**
**Cohort**				
Reference: 1998	1.00	.	1.00	.
2002	0.91 (0.30-2.69)	0.858	1.05 (0.38-2.92)	0.927
2011	0.99 (0.39-2.52)	0.975	1.01 (0.39-2.56)	0.991
**Male**	1.11 (0.48-2.57)	0.811	1.15 (0.50-2.66)	0.735
**Mechanism of injury***				
Reference: Fall	1.00	.	1.00	.
Railway injury	1.96 (0.58-6.55)	0.277	2.03 (0.61-6.74)	0.246
Road traffic injury	1.61 (0.67-3.89)	0.291	1.61 (0.68-3.83)	0.281
**ICISS**	0.94 (0.90-0.98)	0.001	0.94 (0.91-0.98)	0.002

*Assault, other and unknown categories dropped because of too few observations. Abbreviations: CI Confidence Interval, ICD International Classification of Disease, ICISS ICD-derived Injury Severity Score, OR Odds Ratio
