# Supplementary material for: Early Hospital Mortality among Adult Trauma Patients Significantly Declined between 1998-2011: Three Single-Centre Cohorts from Mumbai, India
Source: PLoS One. 2014 Mar 3;9(3):e90064. doi: 10.1371/journal.pone.0090064 (PMC3940776; doi:10.1371/journal.pone.0090064)
Supplement: Table S9 — Multivariate logistic regression model parameters, age 15–55 years analysed separately. (PDF) [file pone.0090064.s009.pdf]

Table S9. Multivariate logistic regression model parameters, age 15-55 years analysed separately

	**Complete case analysis**		**Imputed values**	
	**OR (95% CI)**	**P-value**	**OR (95% CI)**	**P-value**
**Cohort**				
Reference: 1998	1.00	.	1.00	.
2002	0.66 (0.44-0.99)	0.046	0.82 (0.58-1.16)	0.252
2011	0.53 (0.37-0.75)	<0.001	0.52 (0.37-0.74)	<0.001
**Male**	0.98 (0.61-1.57)	0.925	1.00 (0.63-1.58)	0.984
**Mechanism of injury**				
Reference: Fall	1.00	.	1.00	.
Railway injury	3.02 (1.92-4.74)	<0.001	3.08 (1.98-4.80)	<0.001
Road traffic injury	1.47 (0.92-2.34)	0.108	1.50 (0.95-2.37)	0.085
Assault	0.55 (0.24-1.23)	0.147	0.53 (0.24-1.18)	0.119
Other	1.61 (0.36-7.28)	0.533	1.24 (0.28-5.54)	0.775
Unknown	2.84 (1.15-7.01)	0.024	3.07 (1.28-7.35)	0.012
**ICISS**	0.95 (0.94-0.96)	<0.001	0.95 (0.94-0.96)	<0.001

Abbreviations: CI Confidence Interval, ICD International Classification of Disease, ICISS ICD-derived Injury Severity Score, OR Odds Ratio
