# Supplementary material for: Early Hospital Mortality among Adult Trauma Patients Significantly Declined between 1998-2011: Three Single-Centre Cohorts from Mumbai, India
Source: PLoS One. 2014 Mar 3;9(3):e90064. doi: 10.1371/journal.pone.0090064 (PMC3940776; doi:10.1371/journal.pone.0090064)
Supplement: Table S10 — Multivariate logistic regression model parameters, age>55 years analysed separately. (PDF) [file pone.0090064.s010.pdf]

Table S10. Multivariate logistic regression model parameters, age>55 years analysed separately

	**Complete case analysis**		**Imputed values**	
	**OR (95% CI)**	**P-value**	**OR (95% CI)**	**P-value**
**Cohort**				
Reference: 1998	1.00	.	1.00	.
2002	0.34 (0.11-1.09)	0.070	0.39 (0.13-1.21)	0.102
2011	0.43 (0.17-1.10)	0.077	0.42 (0.17-1.07)	0.070
**Male**	2.80 (0.92-8.55)	0.071	2.74 (0.91-8.27)	0.074
**Mechanism of injury***				
Reference: Fall	1.00	.	1.00	.
Railway injury	1.81 (0.54-5.99)	0.334	1.92 (0.60-6.14)	0.271
Road traffic injury	2.31 (0.82-6.51)	0.112	2.16 (0.79-5.89)	0.132
**ICISS**	0.95 (0.91-0.99)	0.011	0.94 (0.91-0.98)	0.002

*Assault, other and unknown categories dropped because of too few observations. Abbreviations: CI Confidence Interval, ICD International Classification of Disease, ICISS ICD-derived Injury Severity Score, OR Odds Ratio
