# Supplementary material for: Early Hospital Mortality among Adult Trauma Patients Significantly Declined between 1998-2011: Three Single-Centre Cohorts from Mumbai, India
Source: PLoS One. 2014 Mar 3;9(3):e90064. doi: 10.1371/journal.pone.0090064 (PMC3940776; doi:10.1371/journal.pone.0090064)
Supplement: Table S11 — Multivariate logistic regression model parameters, patients with fall analysed separately. (PDF) [file pone.0090064.s011.pdf]

Table S11. Multivariate logistic regression model parameters, patients with fall analysed separately

	**Complete case analysis**		**Imputed values**	
	**OR (95% CI)**	**P-value**	**OR (95% CI)**	**P-value**
**Cohort**				
Reference: 1998	1.00	.	1.00	.
2002	0.72 (0.30-1.74)	0.464	0.86 (0.40-1.87)	0.710
2011	1.10 (0.54-2.21)	0.798	1.07 (0.53-2.14)	0.856
**Male**	1.29 (0.57-2.94)	0.538	1.46 (0.65-3.30)	0.359
**Age**				
Reference: <15	1.00	.	1.00	.
15-55	0.72 (0.34-1.50)	0.380	0.68 (0.33-1.42)	0.306
>55	1.19 (0.44-3.25)	0.730	1.16 (0.43-3.10)	0.775
**ICISS**	0.96 (0.93-0.99)	0.012	0.96 (0.93-0.98)	0.001

Abbreviations: CI Confidence Interval, ICD International Classification of Disease, ICISS ICD-derived Injury Severity Score, OR Odds Ratio
