# Supplementary material for: Early Hospital Mortality among Adult Trauma Patients Significantly Declined between 1998-2011: Three Single-Centre Cohorts from Mumbai, India
Source: PLoS One. 2014 Mar 3;9(3):e90064. doi: 10.1371/journal.pone.0090064 (PMC3940776; doi:10.1371/journal.pone.0090064)
Supplement: Table S12 — Multivariate logistic regression model parameters, patients with railway injury analysed separately. (PDF) [file pone.0090064.s012.pdf]

Table S12. Multivariate logistic regression model parameters, patients with railway injury analysed separately

	Complete case analysis		Imputed values	
	**OR (95% CI)**	**P-value**	**OR (95% CI)**	**P-value**
Cohort				
Reference: 1998	1.00	.	1.00	.
2002	0.71 (0.40-1.24)	0.227	0.88 (0.55-1.41)	0.597
2011	0.40 (0.24-0.66)	<0.001	0.39 (0.24-0.64)	<0.001
**Male**	0.95 (0.49-1.84)	0.877	0.94 (0.50-1.78)	0.852
**Age**				
Reference: <15	1.00	.	1.00	.
15-55	1.06 (0.36-3.09)	0.918	1.03 (0.36-2.97)	0.960
>55	1.28 (0.35-4.75)	0.709	1.29 (0.35-4.80)	0.706
**ICISS**	0.94 (0.92-0.96)	<0.001	0.94 (0.92-0.95)	<0.001

Abbreviations: CI Confidence Interval, ICD International Classification of Disease, ICISS ICD-derived Injury Severity Score, OR Odds Ratio
