# Supplementary material for: Early Hospital Mortality among Adult Trauma Patients Significantly Declined between 1998-2011: Three Single-Centre Cohorts from Mumbai, India
Source: PLoS One. 2014 Mar 3;9(3):e90064. doi: 10.1371/journal.pone.0090064 (PMC3940776; doi:10.1371/journal.pone.0090064)
Supplement: Table S13 — Multivariate logistic regression model parameters, patients with road traffic injury analysed separately. (PDF) [file pone.0090064.s013.pdf]

Table S13. Multivariate logistic regression model parameters, patients with road traffic injury analysed separately

	Complete case analysis		Imputed values	
	**OR (95% CI)**	**P-value**	**OR (95% CI)**	**P-value**
**Cohort**				
Reference: 1998	1.00	.	1.00	.
2002	0.56 (0.31-1.01)	0.055	0.68 (0.41-1.13)	0.136
2011	0.59 (0.36-0.98)	0.043	0.60 (0.36-1.00)	0.048
**Male**	1.42 (0.76-2.66)	0.273	1.36 (0.74-2.49)	0.317
**Age**				
Reference: <15	1.00	.	1.00	.
15-55	0.55 (0.29-1.07)	0.078	0.58 (0.30-1.11)	0.098
>55	1.66 (0.74-3.73)	0.220	1.56 (0.70-3.48)	0.275
**ICISS**	0.95 (0.93-0.97)	<0.001	0.95 (0.94-0.97)	<0.001

Abbreviations: CI Confidence Interval, ICD International Classification of Disease, ICISS ICD-derived Injury Severity Score, OR Odds Ratio
